# Supplementary material for: Phosphatidylinositol 3-phosphate and Hsp70 protect Plasmodium falciparum from heat-induced cell death
Source: eLife. 2020 Sep 25;9:e56773. doi: 10.7554/eLife.56773 (PMC7518890; doi:10.7554/eLife.56773)
Supplement: Supplementary file 1. [file elife-56773-supp1.docx]

**Supplementary File 1.** **Primers for gene cloning.**

| PfRan forward | AGGAGGACAGAAAAATGGATTCACAAGAATATATTCCAC^†^ |
| --- | --- |
| PfRan reverse | ATTTTCAATATCTTCTTCATCAATAGC |
| PfHsp70-1 forward | AGGAGGACAGAAAAATGGCTAGTGCAAAAGGTTC^†^ |
| PfHsp70-1 reverse | ATCAACTTCTTCAACTGTTGG |
| PfAlba1 forward | AGGAGGACAGAAAAATGAAGAAAGATAGAGAACCAATAGAC |
| PfAlba1 reverse | TGATAAAGCTCTTCCACCTC |
| 2xFyve forward | AGGAGGACAGAAAAATGCGAATTGAATTCGAAAGTGAT |
| 2xFyve reverse | ACCGTCGACTTTTGCC |
| PfHsp70-1^LID-^ forward | AGGAGGACAGAAAAATGGC |
| PfHsp70-1^LID-^ reverse | GTTTTCTTCATCTTCTGCTTTG |
| RHR^‡^ forward | CGGTACAAACCCGGAATTCGAGCTCGGACTAATAAAAAAAAAAAACATTAAACAGGACAA |
| RHR reverse | CGAGAGATTGGGTATTAGACCTAGGGATAACAGGGTAATTAATAATACTCCTGAATAATCCCATG |
| sgRNA target site | ATTCATCTTTTCCAGCAAGT |
| LHR^§^ (bp 1180–1752) and the re-codonized region (bp 1753–2034) of PfHsp70-1 | TCTGGTGACCAATCAAATGCTGTCCAAGATTTATTATTATTAGATGTTTGCTCCTTATCATTAGGTTTAGAAACTGCTGGTGGTGTTATGACCAAATTAATTGAAAGAAACACAACCATACCTGCTAAAAAGAGTCAAATCTTTACTACTTATGCTGATAACCAACCAGGTGTCTTAATTCAAGTATATGAAGGTGAAAGAGCCTTAACCAAAGATAACAATTTATTAGGAAAATTTCACTTAGATGGTATTCCACCTGCACCAAGAAAGGTACCACAAATCGAAGTTACATTCGATATCGATGCTAACGGTATCTTAAACGTTACGGCTGTAGAAAAATCCACTGGTAAACAAAACCATATTACAATTACCAACGACAAAGGAAGATTATCTCAAGATGAAATTGATCGTATGGTTAATGATGCTGAAAAATACAAAGCAGAAGATGAAGAAAACAGAAAAAGAATCGAAGCAAGAAACAGCCTTGAAAATTACTGCTATGGAGTTAAAAGCTCATTAGAAGACCAAAAAATTAAAGAAAAATTACAACCAGCTGAAATTGAAACATGTATGAAGACCATAACCACCATCCTCGAATGGCTCGAGAAGAACCAATTAGCCGGGAAGGACGAGTATGAAGCCAAGCAAAAAGAAGCCGAATCCGTGTGTGCCCCCATAATGTCCAAGATCTACCAAGATGCTGCAGGAGCTGCTGGCGGAATGCCTGGAGGAATGCCCGGAGGTATGCCCGGGGGTATGCCTGGAGGAATGAATTTTCCAGGAGGAATGCCAGGAGCTGGTATGCCCGGTAATGCACCAGCAGGAAGTGGACCAACAGTAGAAGAAGTAGATTAA |

^†^The sequences in red represent 5′ overhangs containing Shine–Dalgarno sequence and the yeast consensus sequence for efficient protein translation.

^‡^RHR, right homology region of *pfhsp70-1*.

^§^LHR, left homology region of *pfhsp70-1*.
