# Supplementary material for: Phosphatidylinositol 3-phosphate and Hsp70 protect Plasmodium falciparum from heat-induced cell death
Source: eLife. 2020 Sep 25;9:e56773. doi: 10.7554/eLife.56773 (PMC7518890; doi:10.7554/eLife.56773)
Supplement: Supplementary file 2. [file elife-56773-supp2.docx]

**Supplementary File 2. Reported EC_50_ values of small molecule inhibitors used in this study.**

|  | Concentration tested (nM)^†^ | Reported EC_50_ (nM)^‡^ | Arithmetic mean of reported EC_50_ (nM) |
| --- | --- | --- | --- |
| Wortmannin | 20,000 | 55,000 ([1](#_ENREF_1)) | 55,000 |
| LY294002 | 40,000 | 26,000 ([2](#_ENREF_2)) | 26,000 |
| Lapachol | 40,000 | 18,670 ([3](#_ENREF_3)) | 18,670 |
| Artesunate | 20 | 7.8 ([4](#_ENREF_4))  0.5 ([5](#_ENREF_5))  10.2 ([6](#_ENREF_6))  2.7 ([7](#_ENREF_7))  5.4 ([8](#_ENREF_8))  1.6 ([9](#_ENREF_9)) | 4.7 |
| Atovaquone | 40 | 8.91 ([10](#_ENREF_10))  0.8 ([9](#_ENREF_9))  1.68 ([11](#_ENREF_11))  0.84 ([12](#_ENREF_12))  0.9 ([13](#_ENREF_13)) | 2.6 |
| Pyrimethamine | 200 | 5.29 ([14](#_ENREF_14))  38.6 ([15](#_ENREF_15))  47.4 ([15](#_ENREF_15))  31.5 ([9](#_ENREF_9))  13.8 ([16](#_ENREF_16))  3.9 ([17](#_ENREF_17)) | 23 |

^†^Concentration tested in the live cell confocal microscopy (Figure 3 and Figure 3–figure supplement 1).

^‡^EC_50_ values against *P. falciparum* wild-type strain 3D7.

**REFERENCES**

1. A. M. Tomlins *et al.*, Plasmodium falciparum ATG8 implicated in both autophagy and apicoplast formation. *Autophagy* **9**, 1540-1552 (2013).

2. L. Tawk *et al.*, Phosphatidylinositol 3-phosphate, an essential lipid in Plasmodium, localizes to the food vacuole membrane and the apicoplast. *Eukaryotic cell* **9**, 1519-1530 (2010).

3. I. L. Cockburn *et al.*, Screening for small molecule modulators of Hsp70 chaperone activity using protein aggregation suppression assays: inhibition of the plasmodial chaperone PfHsp70-1. *Biological chemistry* **392**, 431-438 (2011).

4. S. Wittlin *et al.*, In vitro and in vivo activity of solithromycin (CEM-101) against Plasmodium species. *Antimicrobial agents and chemotherapy* **56**, 703-707 (2012).

5. A. Kreidenweiss, P. G. Kremsner, B. Mordmuller, Comprehensive study of proteasome inhibitors against Plasmodium falciparum laboratory strains and field isolates from Gabon. *Malaria journal* **7**, 187 (2008).

6. W. Rutvisuttinunt *et al.*, Optimizing the HRP-2 in vitro malaria drug susceptibility assay using a reference clone to improve comparisons of Plasmodium falciparum field isolates. *Malaria journal* **11**, 325 (2012).

7. A. M. Dondorp *et al.*, Artemisinin resistance in Plasmodium falciparum malaria. *The New England journal of medicine* **361**, 455-467 (2009).

8. Z. Wang *et al.*, In vitro sensitivity of Plasmodium falciparum from China-Myanmar border area to major ACT drugs and polymorphisms in potential target genes. *PloS one* **7**, e30927 (2012).

9. N. Fisher *et al.*, Cytochrome b mutation Y268S conferring atovaquone resistance phenotype in malaria parasite results in reduced parasite bc1 catalytic turnover and protein expression. *The Journal of biological chemistry* **287**, 9731-9741 (2012).

10. J. X. Tong, R. Chandramohanadas, K. S. Tan, High-Content Screening of the Medicines for Malaria Venture Pathogen Box for Plasmodium falciparum Digestive Vacuole-Disrupting Molecules Reveals Valuable Starting Points for Drug Discovery. *Antimicrobial agents and chemotherapy* **62** (2018).

11. L. A. Ingasia *et al.*, Molecular characterization of the cytochrome b gene and in vitro atovaquone susceptibility of Plasmodium falciparum isolates from Kenya. *Antimicrobial agents and chemotherapy* **59**, 1818-1821 (2015).

12. Q. L. Fivelman, I. S. Adagu, D. C. Warhurst, Modified fixed-ratio isobologram method for studying in vitro interactions between atovaquone and proguanil or dihydroartemisinin against drug-resistant strains of Plasmodium falciparum. *Antimicrobial agents and chemotherapy* **48**, 4097-4102 (2004).

13. G. A. Biagini *et al.*, Generation of quinolone antimalarials targeting the Plasmodium falciparum mitochondrial respiratory chain for the treatment and prophylaxis of malaria. *Proceedings of the National Academy of Sciences of the United States of America* **109**, 8298-8303 (2012).

14. L. K. Basco, O. Ramiliarisoa, J. Le Bras, In vitro activity of pyrimethamine, cycloguanil, and other antimalarial drugs against African isolates and clones of Plasmodium falciparum. *The American journal of tropical medicine and hygiene* **50**, 193-199 (1994).

15. N. B. Quashie, H. P. de Koning, L. C. Ranford-Cartwright, An improved and highly sensitive microfluorimetric method for assessing susceptibility of Plasmodium falciparum to antimalarial drugs in vitro. *Malaria journal* **5**, 95 (2006).

16. S. Duffy, V. M. Avery, Plasmodium falciparum in vitro continuous culture conditions: A comparison of parasite susceptibility and tolerance to anti-malarial drugs throughout the asexual intra-erythrocytic life cycle. *International journal for parasitology. Drugs and drug resistance* **7**, 295-302 (2017).

17. S. M. Kiara *et al.*, In vitro activity of antifolate and polymorphism in dihydrofolate reductase of Plasmodium falciparum isolates from the Kenyan coast: emergence of parasites with Ile-164-Leu mutation. *Antimicrobial agents and chemotherapy* **53**, 3793-3798 (2009).
